# Linked transmission chains of imported SARS-CoV-2 variant B.1.351 across mainland France, January 2021

**DOI:** 10.2807/1560-7917.ES.2021.26.13.2100333

**Published:** 2021-04-01

**Authors:** The SARS-CoV-2 variant with lineage B.1.351 clusters investigation team, Members of the SARS-CoV-2 variant with lineage B.1.351 clusters investigation team, Members of the SARS-CoV-2 variant with lineage B.1.351 clusters investigation team, Clémentine Calba, Aurélien Zhu-Soubise, Sarah Mahdjoub-Assaad, Berenice Villegas-Ramirez, Arnaud Tarantola, Delphine Barataud, Lisa King, Esra Morvan, Anne Guinard, Olivier Catelinois, Damien Mouly, Guillaume Spaccaferri, Alexandra Mailles, Sibylle Bernard-Stoecklin, Muriel Beliah-Nappez, Aurélie Misme, Diane Descamps, Amine Si Ali, Valérie Garrait, Mounira Smati-Lafarge, Marilyne Gojon, Lucile Trutt, Blandine De Kerros, Josselin Vincent, Colin Deschanvres, Simon Ribes, Florence Daubresse, Olivier Glass, Valérie Lebaillif, Karine Vilhes, Jacques Izopet, Sylvie Van Der Werf, Sylvie Behillil, Etienne Simon-Lorière, Vincent Enouf

**Affiliations:** 1 The members of the team are listed under the Investigator tab and at the end of the article

**Keywords:** COVID-19, SARS-CoV-2 variants 20H/501Y.V2, Cluster, Incubation, Epidemiology

## Abstract

Two cases of confirmed SARS-CoV-2 infection with the B.1.351 variant were reported in France in mid-January, 2020. These cases attended a gathering in Mozambique in mid-December 2020. Investigations led to the identification of five imported cases responsible for 14 transmission chains and a total 36 cases. Epidemiological characteristics seemed comparable to those described before the emergence of the South African variant B.1.351. The lack of tertiary transmission outside of the personal sphere suggests that distancing and barrier measures were effective.

On 13 and 14 January 2021, two cases of confirmed severe acute respiratory syndrome coronavirus 2 (SARS-CoV-2) infection with the B.1.351 variant [1] (or 20H/501Y.V2 in the Nextstrain classification [2]) were reported by the French National Reference Laboratory (NRL) for respiratory diseases to the respective regional health agencies (ARS, regional Ministry of Health’s bureaus) for Ile-de-France and for Pays de la Loire and to Santé publique France (the French public health agency).

These cases had travelled in mid-December 2020 with a group to Mozambique, which shares a border with South Africa, where they participated in a religious gathering. A joint team of epidemiologists, public health workers and clinical and virological specialists cooperated across France to urgently investigate and initiate control measures.

## Definitions

In this investigation, a confirmed case of infection with SARS-CoV-2 variant B.1.351 was any person with a positive test for this variant (specific RT-qPCR or sequencing of the virus genome). A probable case was any returnee from Mozambique with laboratory-confirmed SARS-CoV-2 infection or any person with confirmed SARS-CoV-2 infection and with ascertained at-risk contact with a confirmed case within 14 days before symptom onset or before a positive test. A possible case was defined as any person with ascertained at-risk contact with a confirmed case within 14 days before symptom onset and presenting clinical signs compatible with coronavirus disease (COVID-19). At-risk contact individuals were those with unprotected direct contact with a confirmed case within 1 m or in a confined environment for more than 15 min.

A cluster was defined as at least three cases occurring over a period of 7 days that belonged to the same community or shared the same gathering. A transmission chain was defined as at least two cases over a period of 7 days that shared an epidemiological link.

## Laboratory techniques

First-line positive-RT-qPCR nasopharyngeal samples were forwarded to the National Reference Laboratory for viral genome sequencing and/or to selected reference laboratories of Paris Hospitals (AP-HP) and/or to the University Hospital in Toulouse, France where a specific RT-qPCR was used to identify the N501Y and E484K mutations (TIB Molbiol, Berlin, Germany, and Thermofisher, Waltham, United States (US)).

Samples were processed for whole-genome amplification at the NRL using a highly multiplexed PCR amplicon approach using the ARTIC Network multiplex PCR primers set v3 (https://artic.network/ncov-2019). Dual-indexed sequencing libraries were prepared using Illumina Nextera XT DNA Library Preparation Kit and sequenced using 2 × 150 paired end reads on a NextSeq500 (Illumina, San Diego, United States) on the Mutualized Platform for Microbiology at Institut Pasteur.

## Ethical statement

As per approval by the National Ethical Committee, the ARS and SPF have continual access to personal data in order to investigate and control identified public health threats. No additional ethical clearance was needed or sought. Personal information was anonymised in this publication.

## Epidemiological investigations

The investigations were conducted by the ARS contact tracing teams with the assistance of Santé Publique France. The activities of all cases from 14 days before symptom onset (or before testing date with a positive result) until isolation were mapped to establish potential exposures and identify chains of transmission.

**Figure 1:**
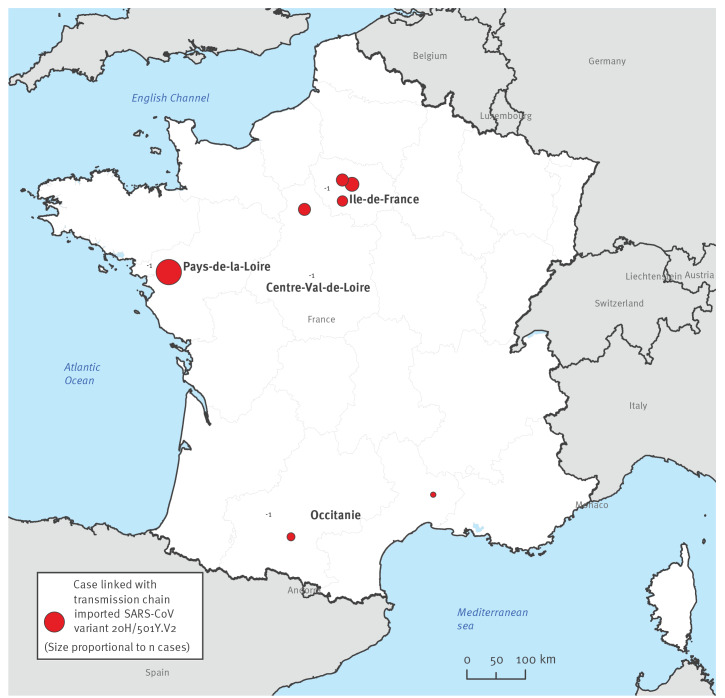
French regions where the clusters and transmission chains were investigated, imported SARS-CoV-2 variant B.1.351, France, January 2021 (n = 36 cases)

A total of five cases were identified who had travelled together: two returned to the west of France (Pays de la Loire region), two to the south (Occitanie region) and one to the Paris region (Ile-de-France region) (Figure 1).

SARS-CoV-2: severe acute respiratory syndrome coronavirus 2.

## Case description and attack rates

All five returnees were RT-qPCR positive for SARS-CoV-2 and two were infected with SARS-CoV-2 variant B.1.351. All were women aged 41–57 years. Two presented symptoms and were hospitalised. Their identified risk factors for severe COVID-19 were hypertension and obesity.

Among the 43 at-risk contacts identified, 41 were tested (two young children were not tested) of whom 31 had positive test results (18 confirmed and 13 probable cases) (Table). The five imported cases gave rise to 14 identified chains of transmission, involving 20 secondary cases (R_eff_ = 4) and 11 tertiary cases (R_eff_ = 1.37). The secondary attack rate (confirmed or probable cases) was estimated at 76.9% and the tertiary attack rate was estimated at 73.3%. Two returnees were involved in superspreading events: in Pays de la Loire (six secondary and six tertiary cases) and in Ile-de-France (11 secondary and five tertiary cases). None of the returnees nor their contacts had been vaccinated against COVID-19.

**Table:** Regional and national epidemiological data of SARS-CoV-2 variant B.1.351 clusters, France, December 2020–January 2021 (n = 36 cases)

	Ile-de-France	Pays de la Loire	Occitanie	Centre-Val de Loire ^a^	Total (national level)
Number of Mozambique returnees	1	2	2	0	5
Known earliest Ct value	20	19; 15	Not available	Not applicable	Not applicable
Households with contacts	6	4	3	1^a^	14
Identified contacts	13	18	6	6	43
Tested contacts	13	18	6	4	41
Confirmed cases among contacts	8	10	0	0	18
Probable cases among contacts	3	3	3	4	13
Undetermined result among contacts	1	0	0	0	1
Negative result among contacts	1	5	3	0	9
Overall attack rate^b^ among tested contacts	11/13	13/18	3/6	4/4	31/41
Secondary attack rate^b^ among tested contacts	7/8	7/11	2/3	4/4^a^	20/26
Tertiary attack rate^b^ among tested contacts	4/5	6/7	1/3^a^	0/0	11/15
Symptomatic cases among positive^b^ contacts	3/11	6/13	1/3	3/4	13/31
Symptomatic cases admitted to hospital	0/3	1/6	0/1	0/3	1/13
Hospitalised cases admitted to intensive care	0/0	0/1	0/0	0/0	0/1
Linked positive tests around the contacts	0/303	0/345	0/2	0/0	0/650

SARS-CoV-2: severe acute respiratory syndrome coronavirus 2.

^a^ Part of the Ile-de-France cluster.

^b^ Confirmed and probable cases.

Among the 31 confirmed and probable secondary and tertiary cases, 17 were women. The median age was 28 years (interquartile range (IQR): 15–39; range: 12–66), with 11 cases younger than 15 years and 14 cases aged 15–30 years. Among all 31 cases, 13 confirmed and probable cases presented symptoms. Symptoms were documented in two of the 11 cases younger than 15 years and in five of the 14 cases aged 15–30 years. One probable case required admission to hospital. The incubation period could be adequately documented in 10 confirmed or probable symptomatic cases, with a median incubation time of 4.5 days (IQR: 2–7; range: 2–10). The return date of one imported case was used to define the at-risk contact date of three symptomatic cases sharing the same household.

## Clusters and chains of transmission

**Figure 2:**
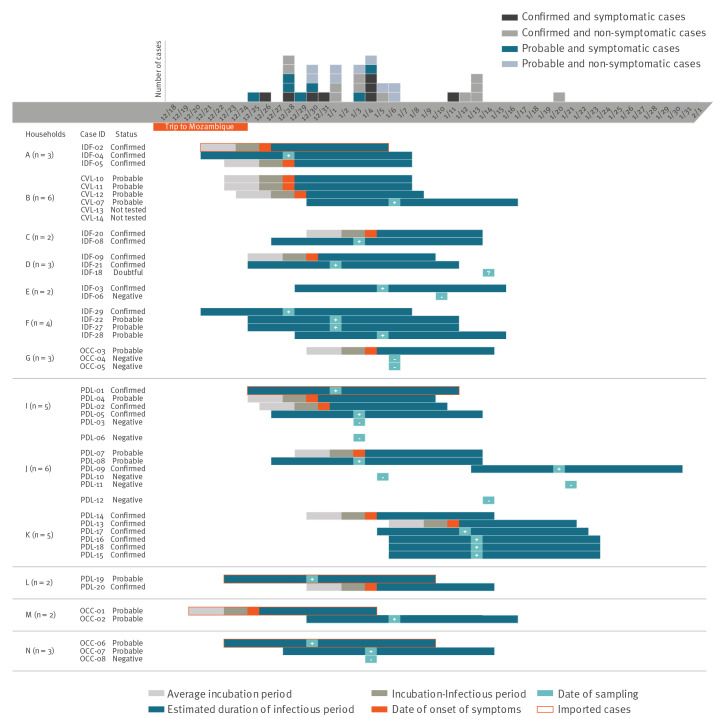
Schematic representation of clusters and chains of transmission of the SARS-CoV-2 B.1.351 variant, France, December 2020–January 2021 (n = 36 cases, 12 at-risk contacts)

The returnee to the Ile-de-France region participated in a family gathering end of December 2020 with individuals from four different households. This gathering took place indoors over a period of 5 h without proper barrier measures. Another individual met with the returnee but did not participate in the gathering. Further investigations identified eight additional at-risk contacts living in three households (including one previously identified). In total, this transmission chain contained 22 at-risk contacts in seven households (five in Ile-de-France, one in Centre-Val de Loire and one in Occitanie). Nineteen were tested and 16 had positive test results (eight confirmed and eight probable cases), one child had undetermined results (Figure 2).

SARS-CoV-2: severe acute respiratory syndrome coronavirus 2.

The two fellow travellers who returned to the Pays de la Loire region were from different families and households. One at-risk contact sharing the same household as one of the two imported cases had a positive test result (confirmed case). For the other imported case, 10 at-risk contacts living in two households were identified. Further investigations identified seven at-risk contacts in two households. In total, 18 at-risk contacts were identified in five households: all were tested and 13 were positive (10 confirmed and three probable cases).

Two fellow travellers returned to the Occitanie region, each giving rise to one secondary case in their respective households. Another case not related to these two travellers but linked to the Ile-de-France cluster, occurred in a third household.

## Control measures

Public health authorities put in place reinforced control measures as soon as the clusters were identified. All cases initiated home quarantine (7–14 days after symptom onset or date of sampling) as soon as they became aware of their own status as cases or of their identification as members of a cluster. No new cases were reported after control measures were implemented around at-risk individuals. Screening of contacts in schools, workplaces and hospitals was also implemented. No related cases were identified in schools (247 and 290 contacts tested in Ile-de-France and Pays de la Loire, respectively), work places (55 contacts tested in Pays de la Loire) or in hospitals where confirmed and probable cases were admitted (56 and two contacts tested in Ile-de-France and Occitanie, respectively).

## Discussion

A new SARS-CoV-2 variant was identified in South Africa in October 2020 [3]. This variant of lineage B.1.351 displays several mutations in the S gene (including N501Y and E484K [3-5]) associated with higher transmissibility and immune escape [3,6].

The overall attack rate among tested contacts of the five imported cases was high (75.6%) but close to that described among several families sharing a household in the French Alps (75–82%) [7]. This was mainly due to familial contacts interacting in a confined indoor space, which may have favoured transmission. Although documented in only 10 symptomatic cases, the median incubation time in our investigation was 4.5 days (IQR: 2–7). This is consistent with previous findings of an incubation period estimated between 4 (IQR: 2–7) [8] and 5.1 days (95% confidence interval: 4.5–5.8) [9] for strains circulating before the SARS-CoV-2 variant B.1.351. The incubation period observed in our clusters could be biased by a limited number of cases, a high percentage of asymptomatic infection and the viral load. Moreover, the reported dates of exposure or symptom onset may not all be accurate owing to recall bias or other challenges in the cluster investigations.

If a sample was positive and epidemiologically associated with a cluster then there was no need for further sequencing according to national guidelines at the time; therefore, some of the samples were not sequenced. Nonetheless, sequencing these samples could have brought additional information that could have led to the identification of further mutations of interest. Systematic nasopharyngeal RT-qPCR among contacts younger than 6 years is not recommended in France. Limits of nasopharyngeal samples among young children and the context of delayed investigations raise the issue of the potential use of other anatomical sites that can be used for testing, such as saliva or stools.

Half of the cases described here resulted from two family clusters involving superspreading events. Before the emergence of the new variants, the major role that these events played in SARS-CoV-2 transmission had been described in detail [10,11]. No cases were identified outside of the households involved in these events. Because of this specific familial transmission context, epidemiological indicators calculated based on this investigation cannot easily be extrapolated to the general population. Moreover, superspreading events are not the best models to understand epidemiological features in the population but rather worst-case scenarios with prolonged exposure in confined spaces [11-13]. In addition, the family cluster in Ile-de-France draws the attack rate upwards, thereby suggesting a potential role of familial vulnerability genetic profiles, as is the case with other respiratory pathogens [14].

Another challenge was that some members of the clusters did not agree to answer questions, highlighting the need for epidemiologists and public health workers capable of convincing interviewees to share information of public health importance.

Despite these limitations, this investigation provided an invaluable opportunity to determine incubation time, transmission rate, proportion of asymptomatic cases, pre-symptomatic transmission, clinical spectrum and severity of SARS-CoV-2 variant B.1.351. The absence of identified tertiary transmission in schools, hospitals or workplaces denotes the apparent effectiveness of distancing and barrier measures, despite the likelihood of a more transmissible B.1.351 variant.

## Conclusion

Religious and other large gatherings have played a significant role in SARS-CoV-2 spread since 2020 [15-18]. Imported cases serve as sentinel events for affected countries which are unable to monitor emerging variants. At the time of the investigation, the SARS-CoV-2 B.1.351 variant had only been reported in South Africa. This highlights the difficulties in establishing guidelines to identify at-risk areas, which was already a challenge in the early phase of the pandemic. Before this investigation, there may have been other imported cases of variant B.1.351, which were not detected.

According to the limited data yielded by this investigation, incubation times and clinical attack rates of this variant seem in line with those described for the previously circulating SARS-CoV-2 strains. Intervals, case fatality rates and reproductive numbers in communities, especially by age group and sex at birth, must be better documented to guide further public health actions. Messages on barrier and distancing measures should be reinforced.

## The SARS-CoV-2 variant with lineage B.1.351 clusters investigation team

Clémentine Calba, Aurélien Zhu-Soubise, Sarah Mahdjoub-Assaad, Berenice Villegas-Ramirez, Arnaud Tarantola, Delphine Barataud, Lisa King, Esra Morvan, Anne Guinard, Olivier Catelinois, Damien Mouly, Guillaume Spaccaferri, Alexandra Mailles, Sibylle Bernard-Stoecklin (Santé publique France, Saint-Maurice, France); Muriel Beliah-Nappez, Aurélie Misme (Agence régionale de santé Ile-de-France, Saint-Denis, France); Diane Descamps (Service de Virologie, Université de Paris, Hopital Bichat-Claude Bernard AP-HP Nord, INSERM UMR 1137 IAME, équipe DESCID, Paris, France); Amine Si Ali (Unité Contrôle-Prevention des Infections (CPI), CH Inter Communal de Créteil, Créteil, France); Valérie Garrait (Médecine interne, CH Inter Communal de Créteil, Créteil, France); Mounira Smati-Lafarge (Laboratoire de Biologie Médicale, CH Inter Communal de Créteil, Créteil, France); Marilyne Gojon, Lucile Trutt, Blandine De Kerros, Josselin Vincent (Agence régionale de santé Pays de la Loire, Nantes, France); Colin Deschanvres, Simon Ribes (Centre Hospitalier Universitaire de Nantes, Nantes, France); Florence Daubresse, Olivier Glass, Valérie Lebaillif, Karine Vilhes (Agence régionale de santé Occitanie, Toulouse, France); Jacques Izopet (Laboratoire de Virologie, Institut Fédératif de Biologie, Toulouse, France); Sylvie Van Der Werf, Sylvie Behillil (Unité de Génétique Moléculaire des Virus à ARN, Institut Pasteur, UMR 3569 CNRS, Université de Paris, Centre National de Référence Virus des Infections Respiratoires, Paris, France); Etienne Simon-Lorière (G5 Evolutionary Genomics of RNA Viruses, Institut Pasteur, Paris, France); Vincent Enouf (Unité de Génétique Moléculaire des Virus à ARN, Institut Pasteur, UMR 3569 CNRS, Université de Paris, Centre National de Référence Virus des Infections Respiratoires, Paris, France ; Plateforme de Microbiologie Mutualisée (P2M), Pasteur International Bioresources network (PIBnet), Institut Pasteur, Paris, France).
